# Intra-articular Hyaluronic Acid for Knee Osteoarthritis: Stabilizing Utilization Trends Amid Conflicting Clinical Practice Guidelines

**DOI:** 10.2106/JBJS.OA.25.00335

**Published:** 2026-01-16

**Authors:** Chris J. Lee, Albert H. Lee, Wesley Day, Jonathan N. Grauer

**Affiliations:** 1 Yale School of Medicine, Department of Orthopaedics & Rehabilitation, New Haven, Connecticut

## Abstract

**Background:**

Evidence has been mixed about the efficacy of intra-articular hyaluronic acid (HA) for knee osteoarthritis. This has led to conflicting clinical practice guidelines (CPGs) over the years. After the American Academy of Orthopaedic Surgeons (AAOS) issued a strong recommendation against HA in 2013, a claims-based study showed rapid decline in use. More recent endorsements from Osteoarthritis Research Society International (OARSI) in 2019 and the Veterans Affairs and Department of Defense (VA-DoD) in 2020 may have altered this trajectory. This study aimed to gauge contemporary utilization of HA knee injections.

**Methods:**

All patients aged 18 years and older diagnosed with knee osteoarthritis were identified from the 2010Q1-2023Q1 PearlDiver database. The percentage of patients receiving intra-articular HA relative to the number of patients diagnosed for knee osteoarthritis was calculated quarterly. Linear regression analyses were segmented by 2 key CPG inflection points: 2013Q3 AAOS' recommendation against HA injections and the 2019Q4 endorsements. Analyses were also stratified by provider specialty. Statistical significance was set at p < 0.05.

**Results:**

A total of 16,581,526 knee OA patients were identified, among which HA knee injections were performed for 1,886,788 (11.4%). For the post-2013 AAOS CPG period (2013Q3-2019Q3), injection rates decreased (–0.10% per quarter; p < 0.001). However, following OARSI/VA-DoD endorsement (2019Q4-2023Q1), the slope leveled to –0.003% per quarter; p = 0.921. Through the study period, utilization declined for both women and men and both younger and older patients (<50 years old and ≥ 50 years old) (p < 0.001 for all). Utilization declined among orthopaedic surgeons, nonoperative musculoskeletal specialists, and primary care physicians, while utilization increased among pain medicine physicians (p < 0.001 for all).

**Conclusions:**

Intra-articular HA injection use decreased after the 2013 CPG from AAOS but has stabilized after more positive 2019 and 2020 CPGs from OARSI/VA-DoD. Notably, practice patterns are diverging patterns across specialties, suggesting variabilities in use.

**Level of Evidence:**

Level III. See Instructions for Authors for a complete description of levels of evidence.

## Introduction

Knee osteoarthritis (OA) affects over 14 million Americans and remains a leading cause of pain, disability, and healthcare spending^1,2^. Among the nooperative treatment options, intra-articular hyaluronic acid (HA) injections are widely used, and it has been reported that 14.7% of patients with knee OA receive viscosupplementation within 12 months before total knee arthroplasty^3^. Despite widespread usage, the efficacy of HA injections has conflicting and limited evidence^4-11^.

Clinical practice guidelines (CPGs) have evolved over time and mirrored this uncertainty (Table I). The first American Academy of Orthopaedic Surgeons (AAOS) guideline (2008) was unable to make a recommendation for or against the use of HA, marking it as inconclusive^12^. However, a re-evaluation in the 2013 second edition AAOS guideline reversed course with a strong recommendation against its use^13^. According to Bedard et al. (2018), a study based on the 2007 to 2015 Humana claims database, the previously rising rate of HA injections flipped to a decline of –0.12 injections per 100 knee-OA patients each quarter following the publication of this second edition CPG in 2013 and potentially increased difficulty in approval^14^. This suggested that CPGs could indeed influence nationwide clinical decisions.

**TABLE I:** Summary of Knee Osteoarthritis Clinical Practice Guidelines on Intra-articular Hyaluronic Acid Injections

Guideline Publisher	Year Published	HA injection	Publication
AAOS	2008	Unable to make a recommendation for or against	*Treatment of Osteoarthritis of the Knee (nonarthroplasty), 1st ed. 2008Q4*
AAOS	2013	Strong recommendation against use	*Treatment of Osteoarthritis of the Knee, 2nd ed. 2013Q3*
OARSI	2019	Conditional/appropriate for knee; not recommended for hip/poly-osteoarthritis	*Nonsurgical Management of Knee, Hip & Polyarticular OA, 2019Q4*
ACR	2019 (published 2020)	Conditional recommendation against (knee); strong against (hip)	*ACR/AF Guideline for the Management of OA of the Hand, Hip and Knee, 2020Q1*
US VA/DoD	2020	Conditional recommendation for selected patients (“viscosupplementation”)	*Nonsurgical Management of Hip & Knee OA, 2020Q3*
AAOS	2021	Not recommended for routine use	*Management of Osteoarthritis of the Knee (Non-Arthroplasty), 3rd ed. 2021Q3*
UK NICE	2022	Do not offer hyaluronan injections	*NG226 Osteoarthritis in over 16 s: diagnosis and management, 2022Q3*

AAOS = American Academy of Orthopaedic Surgeons, ACR = American College of Rheumatology, HA = hyaluronic-acid, NICE = National Institute for Health & Care Excellence, OARSI, Osteoarthritis Research Society International, and VA/DoD = US Veterans Affairs & Defense.

However, newer CPGs related to HA injections for knee OA have been more mixed. The Osteoarthritis Research Society International (OARSI) in 2019 and the US Veterans Affairs/Department of Defense (VA-DoD) in 2020 both issued conditional endorsements for HA in knee-OA patients^15,16^. However, guidelines from AAOS, American College of Rheumatology, and the United Kingdom’s National Institute for Health & Care Excellence each recommended against intra-articular HA usage in guidelines newly published in 2021, 2020, and 2022, respectively^17-19^. As a result, clinicians have been forced to navigate their clinical decisions amid conflicting statements from authoritative bodies.

Despite these developments, no study has examined how these newer guidelines have influenced utilization trends in HA injections for knee OA. Several questions remain as to whether the initial post-2013 downturn has persisted, plateaued, or reversed, and whether utilization is following different trends in different patient demographics and provider specialties. Previous studies either ended before 2019, focused on a single payer, or lacked specialty-level detail, leaving a contemporary evidence gap^14,20,21^.

The hypothesis behind this study is that inflection in attitudes of CPGs toward intra-articular HA injections alter utilization trends with more recent positive CPGs leading to reversal from downward trend in utilization. The purpose of this analysis was to clarify how usage patterns have evolved in the face of conflicting guidelines pertaining to HA injections for knee OA.

## Materials and Methods

### Data Source and Patient Selection Criteria

Data were used from the 2010Q1 to 2023Q1 PearlDiver M170 national database (PearlDiver Technologies, Colorado Springs, CO). This database contains 170 million patient records from multiple insurances and is well-utilized in studying utilization trends of orthopaedic treatments^22-25^. A previous version of this database was used by the previous paper studying HA utilization trends for knee OA^14^. Our Institutional Review Board determined studies using this database to be exempt from review.

The database was queried for all patients aged 18 years or older coded for knee OA by International Classification of Disease (ICD) coding. Those who had HA injections were determined by C, J, and Q Healthcare Common Procedure Coding System (HCPCS) codes^14,20^. Only injections marked with a same-day ICD-9 or ICD-10 diagnosis of knee OA were included.

For each quarter from 2010Q1 to 2023Q1, the percentage of patients receiving a HA injection among those with a knee OA diagnosis was calculated. Quarters were defined as the following dates: Q1 (January 1-March 31), Q2 (April 1-June 30), Q3 (July 1-September 30), and Q4 (October 1-December 31). Patients billed for multiple injections during a single quarter were only accounted for once, as the focus was patient count rather than number of injections. For subgroup analyses, trends were also analyzed based on sex (male versus female) and younger patients (<50 years) and older patients (≥50 years).

### Provider Specialties

Provider specialty codes were then abstracted to evaluate specialty-specific utilization trends. The 4 broad categories of physician specialties were orthopaedic surgeons, nonoperative musculoskeletal specialists, pain specialists, and primary care physicians. This classification system was adopted from a previously used by a study analyzing utilization trends of HA injections^14^.

Nonoperative musculoskeletal specialists included rheumatologists, physical medicine and rehabilitation, and internal medicine sports medicine. Pain specialists included providers in pain medicine and anesthesiology. Primary care physicians included physicians of the following specialties: family medicine, internal medicine, emergency medicine, and general practice. Injections by physicians not conforming to these 4 categories were excluded from this part of the analysis, as were injections where provider specialty codes were not available.

### Data Analyses

Utilization trends were plotted as percentage of knee OA patients that received HA injections per quarter. First, trends were assessed using regression to show changes in utilization rates over the entire study period from 2010Q1 to 2023Q1. Second, to evaluate the potential correlations of the conflicting CPGs, an interrupted time series analysis with segmented regression was conducted to separately analyze each period. The study period was divided into 3 major segments: pre-2013 AAOS CPG (2010Q1-2013Q2), post-2013 AAOS CPG (2013Q4-2019Q3), and after the OARSI/VA-DoD CPG (2020Q1-2023Q1). This approach allowed both a broader view on overall trends and quantification of the impact of CPGs in detail.

Regression models accounted for seasonality via Seasonal and Trend decomposition using Loess and autocorrelation using Newey-West standard errors. To quantify seasonal effects, estimated marginal means were used to perform both comparisons between quarters.

All statistical analyses were conducted in R version 4.4.2 (R Foundation for Statistical Computing). Utilization trends were reported as changes in percentage points per quarter. Seasonal differences were reported as relative percentage differences estimated marginal means, calculated by dividing the difference between quarters by the reference quarter's mean. Statistical significance for this study was established at p < 0.05.

## Results

### Study Population

A total of 16,581,526 adult patients diagnosed with knee OA were identified, of which HA injection was performed for 1,886,788 (11.4%) (Table II). Female patients accounted for 10,427,135 (63%) of the knee OA study population, with HA injection performed for 1,218,748 (11.7%) of female patients. Male patients accounted for 6,154,391 (37%) of the knee OA study population, with HA injection performed for 668,040 (10.9%) of male patients. Although used across all age ranges, when stratified by decades of age, patients younger than 30 years had the lowest utilization rate (5.2%), while those aged 70 years and older had the highest (11.3%).

**TABLE II:** Demographic Breakdown of Hyaluronic Acid Utilization Among Patients Diagnosed with Knee Osteoarthritis

	Total			Pre-2013 AAOS CPG (2010Q1-2013Q2)			Post-2013 AAOS CPG (2013Q4-2019Q3)			Post-OARSI/VA-DoD CPG (2020Q1-2023Q1)		
	No. of Patients with Knee Osteoarthritis	Received Hyaluronic Acid		No. of Patients with Knee Osteoarthritis	Received Hyaluronic Acid		No. of Patients with Knee Osteoarthritis	Received Hyaluronic Acid		No. of Patients with Knee Osteoarthritis	Received Hyaluronic Acid	
		No	%		No	%		No	%		No	%
Total	16,581,526	1,886,788	11.4	5,368,754	611,866	11.4	9,743,384	962,389	9.9	5,893,332	490,835	8.3
Sex												
Female	10,427,135	1,218,748	11.7	3,424,686	395,555	11.6	6,177,189	621,627	10.1	3,777,655	318,634	8.4
Male	6,154,391	668,040	10.9	1,944,068	216,311	11.1	3,566,098	340,754	9.6	2,115,654	172,200	8.1
Age in years*												
<30	137,065	7,112	5.2	43,479	2,885	6.6	67,956	3,098	4.6	26,477	1,061	4.0
30-34	191,909	11,891	6.2	56,964	4,417	7.8	96,635	5,563	5.8	39,734	1,854	4.7
35-39	369,443	27,018	7.3	104,236	9,292	8.9	190,834	12,978	6.8	82,547	4,893	5.9
40-44	692,164	58,071	8.4	201,824	20,339	10.1	349,195	27,032	7.7	166,905	11,284	6.8
45-49	1,206,865	110,633	9.2	355,416	38,355	10.8	625,326	52,874	8.5	285,445	20,960	7.3
50-54	1,998,482	191,372	9.6	579,811	65,095	11.2	1,037,247	91,203	8.8	504,126	38,499	7.6
55-59	2,753,837	263,645	9.6	757,979	85,113	11.2	1,454,196	129,228	8.9	738,502	55,545	7.5
60-64	3,221,866	302,099	9.4	842,967	92,467	11.0	1,674,469	147,311	8.8	939,603	69,413	7.4
65-69	3,329,513	345,336	10.4	862,040	99,899	11.6	1,686,974	167,598	9.9	1,014,510	86,367	8.5
≥70†	6,753,509	764,950	11.3	1,939,027	221,939	11.4	3,754,168	388,561	10.4	2,482,693	220,196	8.9

* If a patient had received an injection at multiple times during the study period and his or her age at the time of a given injection fell within different age groups, then the patient was captured multiple times in the age group breakdown.

† The ≥70 category includes everyone aged 70 years and older due to patient privacy protections by the database.

### Utilization Trends by Period and by Patient Demographics

**Fig. 1:**
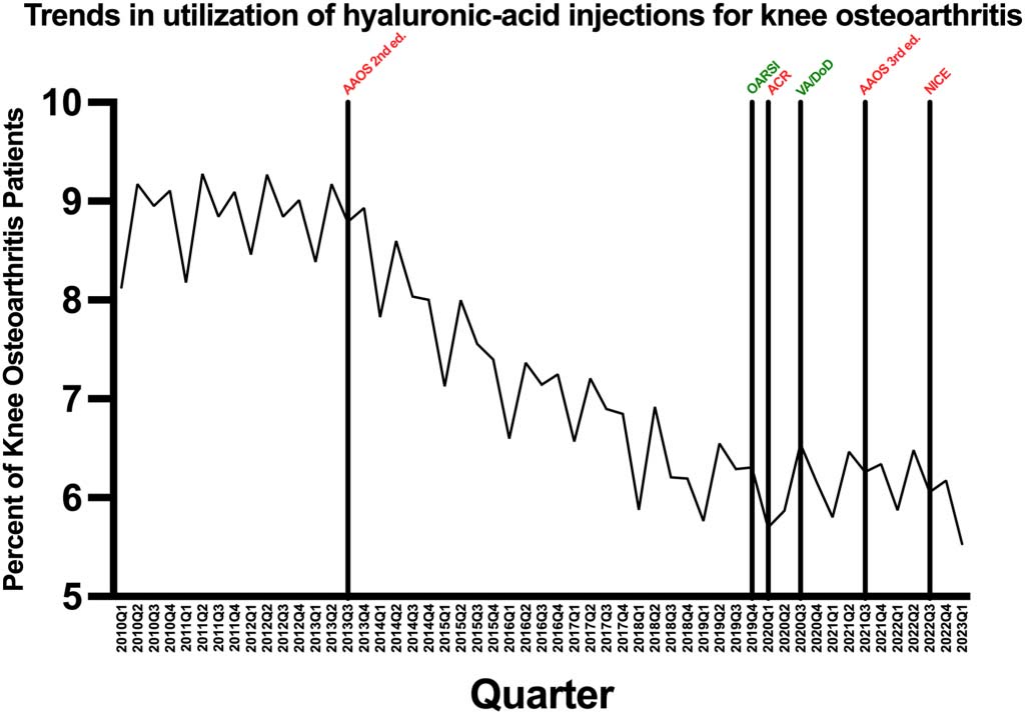
Utilization of intra-articular HA injections among patients evaluated for knee osteoarthritis each quarter from 2010Q1 to 2023Q1. Vertical lines indicate major CPG publications; green = pro-HA, red = against HA. HA = hyaluronic acid.

**Fig. 2:**
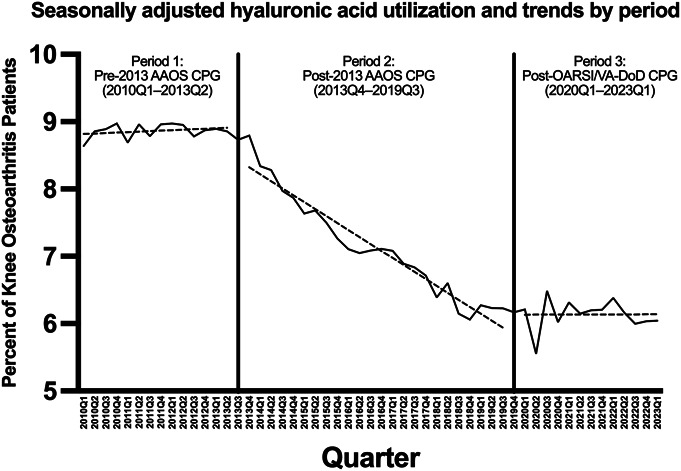
Seasonally adjusted utilization trends in intra-articular HA injection among patients with knee osteoarthritis from 2010Q1 to 2023Q1, segmented by 3 CPG inflection periods. 2010Q1 to 2013Q2: pre-2013 guideline period. 2013Q4 to 2019Q3: after the 2013 AAOS second-edition guideline recommending against HA. 2020Q1 to 2023Q1: following the publication of more pro-HA CPGs from OARSI and VA/DoD in late 2019 and 2020. AAOS = American Academy of Orthopaedic Surgeons, HA = hyaluronic acid, CPG = clinical practice guideline, OARSI, Osteoarthritis Research Society International, and VA/DoD = US Veterans Affairs & Defense.

Overall, intra-articular HA injection utilization rates decreased by 0.07 percentage points per quarter from 2010Q1 to 2023Q1 (p < 0.001) (Fig. 1). Specifically, from 2010Q1 to 2013Q2, which marks the period before the publication of the 2013 AAOS second-edition CPG, the rate of change in utilization was statistically insignificant (+0.01% per quarter; p = 0.324). However, following the publication of the 2013 AAOS CPG, from 2013Q4-2019Q3, HA injection rates decreased by 0.10 percentage points for quarter (p < 0.001). Utilization rates after the publication of OARSI and US Veterans Affairs & Defense (VA/DoD) CPGs leveled off with a statistically insignificant slope in 2020Q1-2023Q1 (–0.0004% per quarter; p = 0.981) (Fig. 2).

Overall, utilization rates among both female and male patients declined 0.07 percentage points per quarter (p < 0.001 for both). Among younger patients aged younger than 50 years, the utilization rate declined at a rate of 0.08 percentage points per quarter (p < 0.001), while usage among patients aged 50 years or older declined 0.07 percentage points per quarter (p < 0.001).

### Provider Specialty

**Fig. 3:**
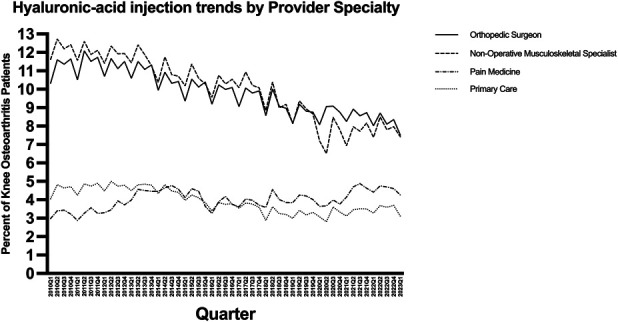
Trends in intra-articular hyaluronic acid injection utilization for knee osteoarthritis from 2010Q1 to 2023Q1, stratified by provider type.

Orthopaedic surgeons accounted for 62.2% of all HA injections, nonoperative musculoskeletal specialists accounted for 9.3%, pain specialists accounted for 3.3%, and primary care physicians accounted for 13.4%. The overall rate of HA injections decreased by 0.07% per quarter for orthopaedic surgeons (p < 0.001), decreased by 0.10% per quarter for nonoperative musculoskeletal specialists (p < 0.001), decreased 0.03% per quarter for primary care physicians (p < 0.001), and increased by 0.02% per quarter for pain specialists (p = 0.004) (Fig. 3).

### Seasonality

Across all knee-OA patients, HA utilization showed a seasonal pattern: utilization consistently dipped in the first quarter. Compared with the rest of the year, Q1 rates were 9.1% lower for all patients (p < 0.001). When compared pairwise to each quarter, Q1 rates were 10.5% lower than Q2 (p < 0.001), 7.7% lower than Q3 (p = 0.002), and 9.0% lower than Q4 (p < 0.001). No other pairwise differences (Q2-Q3, Q2-Q4, and Q3-Q4) were statistically significant. A similar pattern appeared across provider subgroups: relative to the rest of the year, Q1 rates were 9.3% lower among orthopaedic surgeons (p < 0.001), 8.1% lower among nonoperative musculoskeletal specialists (p < 0.001), 8.4% lower among pain specialists (p = 0.010), and 9.7% lower among primary care providers (p < 0.001).

## Discussion

This study examined the contemporary utilization trends of intra-articular HA injections for knee OA across a multipayer, national database. In line with previous studies, this study showed a significant decline in HA use following the 2013 AAOS CPG that recommended against its use^14,20^. However, the study also shows that this decline flattened out after the 2019 to 2020 conditional endorsements by the OARSI and VA/DoD. These results suggest that, while CPGs can meaningfully alter clinical practice, conflicting recommendations may neutralize previous momentum.

Data from previous studies were limited to one type of insurance, such as Humana or Medicare, while this study extracted data from a multipayer claims database that covers patients in all 50 states^14,20,21^. This study is more representative of a nationwide sample. Furthermore, other studies have only reported up to 2018, while this study presents data up to 2023^20^. As a result, this study is the first to evaluate the effect of more current CPGs published from 2019 to 2022 and to incorporate data from 13 years.

CPGs are important in determining clinical practice and decision making of providers and may affect insurance coverage. There are well-cited historical examples where publication of a CPG measurably shifted utilization trends, such as the drop in mammography usage following the US Preventive Services Taskforce's 2009 recommendation against routine mammography in women aged 40 to 49 and the drop in routine episiotomy after the American College of Obstetricians and Gynecologists’ 2006 guideline^26,27^. Specifically for intra-articular HA injections for knee OA, the underlying evidence on the effectiveness of HA is still contested, leading to weak conclusions and conflicting guidelines. Some trials demonstrate a statistically significant benefit in short-term pain relief and stiffness, while others report no meaningful improvement^4-11,28^. The controversy is further complicated by important study-level variables: the formulation, the baseline severity of OA, and the time points at which outcomes are assessed^11,29,30^. This variability in studies and injection practices have made it difficult to fully ascertain the clinical benefit of intra-articular HA for knee OA.

An additional finding in our analysis was the divergence in utilization patterns across provider specialties. HA use decreased among orthopaedic surgeons, nonoperative musculoskeletal specialists, and primary care physicians but increased among pain medicine specialists. This mirrors a previous study showing a decline among orthopaedic surgeons but an increase among physician assistants and nurse practitioners^20^. A potential external factor influencing injection practice may be changing financial pressures within the clinic regarding visit type and providers. Although it is difficult to determine why some medical professionals are trending in the opposite direction, there are several hypothetical reasons that may warrant further exploration. It may be that there are differences in CPG emphases for different specialists, interpretations of existing literature, referral patterns, and expectations of patients. This interspecialty divergence underscores the complexity of aligning practice patterns with evidence, particularly in multidisciplinary fields such as musculoskeletal care.

Seasonality also emerged as a pattern across all provider groups. Utilization dropped in the first quarter of each year—by approximately 9% compared with other quarters. This phenomenon is consistent with prior research on elective medical procedures and is likely a result of the insurance deductibles reset in January^31,32^. Adjusting for these seasonal effects is important for accurate interpretation of utilization trends. Researchers of trends in elective procedures may wish to account for seasonality in their analyses.

This study has several limitations. As with any research based on administrative claims data, our analysis is dependent on accurate coding and documentation. We were unable to assess patient-level clinical details such as disease severity, laterality, or patient-reported outcomes. Some provider specialties may have been misclassified. In addition, changes in HA use may not simply reflect physicians' practice patterns: changes in insurance coverage, marketing, or patient preference may also have played a role.

In conclusion, this study shows that the initial decline in intra-articular HA injections following the 2013 AAOS guideline has plateaued in the context of more recent and supportive HA recommendations. Utilization trends also appear to be shaped by specialty-specific practices. These findings suggest that while guidelines can meaningfully influence clinical behavior, sustained change requires consistency across professional societies. Future work should incorporate clinical outcomes, patient satisfaction, and cost-effectiveness data to better understand when and for whom intra-articular HA injections offer true value in managing knee OA.

## Code Availability

All codes used were either from preexisting codes from the PearlDiver software or custom-made directly in the software. Codes are available on request.

## Availability of Data and Material

All data are available from the national insurance claims database, M170Ortho PearlDiver.
